# The role of advance directives in end-of-life decisions in Austria: survey of intensive care physicians

**DOI:** 10.1186/1472-6939-11-19

**Published:** 2010-10-21

**Authors:** Eva Schaden, Petra Herczeg, Stefan Hacker, Andrea Schopper, Claus G Krenn

**Affiliations:** 1 Department of Anaesthesiology, General Intensive Care and Pain Management Medical University of Vienna, Austria; 2 Department of Journalism und Communication University of Vienna, Faculty of Social Sciences, Austria; 3 Self-employed doctor-of-laws Graz, Austria

## Abstract

**Background:**

Currently, intensive care medicine strives to define a generally accepted way of dealing with end-of-life decisions, therapy limitation and therapy discontinuation.

In 2006 a new advance directive legislation was enacted in Austria. Patients may now document their personal views regarding extension of treatment. The aim of this survey was to explore Austrian intensive care physicians' experiences with and their acceptance of the new advance directive legislation two years after enactment (2008).

**Methods:**

Under the aegis of the OEGARI (Austrian Society of Anaesthesiology, Resuscitation and Intensive Care) an anonymised questionnaire was sent to the medical directors of all intensive care units in Austria. The questions focused on the physicians' experiences regarding advance directives and their level of knowledge about the underlying legislation.

**Results:**

There were 241 questionnaires sent and 139 were turned, which was a response rate of 58%. About one third of the responders reported having had no experience with advance directives and only 9 directors of intensive care units had dealt with more than 10 advance directives in the previous two years. Life-supporting measures, resuscitation, and mechanical ventilation were the predominantly refused therapies, wishes were mainly expressed concerning pain therapy.

**Conclusion:**

A response rate of almost 60% proves the great interest of intensive care professionals in making patient-oriented end-of-life decisions. However, as long as patients do not make use of their right of co-determination, the enactment of the new law can be considered only a first important step forward.

## Background

Advances in therapeutic and technical possibilities further narrow the already thin line between sustaining life and prolonging the process of dying in the intensive care unit (ICU). Thus, intensive care physicians nowadays see themselves increasingly confronted with the need of additionally having to assume responsibilities originally pertaining to palliative care [1].

As recent literature shows, intensive care medicine strives to define a generally accepted way of dealing with end-of-life decisions, therapy limitation and therapy discontinuation [2-4]. Awareness of this delicate subject is also raised by means of terminological modifications - for example "Do Not Resuscitate" orders were further differentiated by adding the word attempt ("Do Not Attempt Resuscitation") or replaced by the term "Allow Natural Death" [5,6].

Anyway, most of all it is essential to ensure the autonomy and dignity of each and every patient. Yet, since many of ICU patients are unconscious or in another way incapable of expressing their wishes, the decision making process often has to be based on the patients' presumed instead of their known will.

For these reasons, in 2006 a new advance directive legislation was enacted in Austria [7]. Supported by medical and legal advice, patients document their personal views regarding extension of treatment, e.g. mechanical ventilation, resuscitation or nutrition. Additionally, they can express their wishes, e.g. concerning pain therapy, within a legal framework. It is important to mention that "Termination of Life on Request" and "Assisted Suicide Act", which have been permitted in the Netherlands since 2002, are still illegal in Austria. The advance directive can be laid down in binding form (doctors must execute the patient's wishes in any case) or in non-binding form (as statements of wishes to be given due respect, i.e. in case of doubt, doctors are allowed to deviate from the given directives).

Key data regarding the Austrian law include:

✓ Law came into force 01/06/2006 (it was the very first time a law of that kind was enacted in Austria).

✓ Patients document their personal views regarding extension of treatment, e.g. mechanical ventilation, resuscitation or nutrition and they can express their wishes, e.g. concerning pain therapy but only in accordance with best clinical practice.

✓ AD forms are available and can be used, but also hand-written paperwork not using specific terminology is valid.

✓ The situation the AD should be applied to has to be described in detail.

✓ The AD can be laid down in a binding or a non-binding form.

✓ Binding AD: The treating physician is obliged to keep to the AD. Therefore, the patient has to obtain advice from a physician (decides whether the patient is capable of decision making, explains different therapies in detail and helps to describe concrete situations) and a notary (for acknowledgement). This procedure may cost up to 500 Euro, i.e. 1/3 of an average Austrian middle class monthly salary. The validity of a binding AD is 5 years, renewal needs again medical and legal advice. If it is not renewed, the status changes to a non-binding AD.

✓ Non binding AD: Meant as a statement of wishes to be given due respect. Can be laid down by the patient on his/her own, does not need any witnesses or acknowledgement, therefore free of cost.

✓ So far there is no nationwide database where doctors can look up for existing ADs. Patients are advised to inform a relative or a representative about the existence and the depository of the AD, so in case they can make it accessible to the treating physician. After creation of a binding AD patients get an AD identification card (like credit card), which they can put into their wallet.

The aim of this survey was to explore Austrian intensive care physicians' experiences with and their acceptance of the new advance directive legislation two years after enactment.

## Methods

Prior to the creation of the questionnaire for the survey we performed some personal interviews with colleagues. The questionnaire was designed in cooperation with a doctor-of-laws and with a publicist whose expertise is communication in medical settings.

In Austria, every ICU is managed by a medical director who, besides his/her administrative duties, is also involved in routine clinical decisions including therapy withholding and/or withdrawal. Therefore, we addressed the questionnaire to the medical directors of all intensive care units in Austria, including anaesthesiological, medical, surgical, neurosurgical, paediatric and neonatal intensive care units as well as intermediate care units. The addresses were taken from the recent registry of the Austrian Chamber of Physicians.

The survey explored the experience physicians had hitherto made with advance directives, and with their level of knowledge about the underlying legislation.

In the first part we asked how many advance directives the physicians had dealt with in the previous 2 years and how they had come to know about the existence of such a directive. The views and wishes their patients had documented in the advance directives had to be listed. We also inquired about the readiness of the physicians of adhering to these advance directives and about the potential conflicts arising from the existence of such a directive.

In the second part of the questionnaire physicians were asked where they obtained information about advance directive legislation, their knowledge about the different kinds of advance directives (binding versus non-binding), and if they knew patients had the right to revoke the directive.

Finally, we wanted to find out, if intensive care physicians consider advance directives to be helpful tools and if they recommend creating an advance directive to their patients. We also asked, if physicians had laid down directives for themselves.

The anonymised questionnaire consisted of 5 pages all together (Additional file 1) and was sent by mail in November 2008 under the aegis of the OEGARI (Austrian Society of Anaesthesiology, Resuscitation and Intensive Care). A post-paid returning envelope was enclosed, a deadline of 4 weeks was deemed appropriate for them to return the questionnaire.

The review board of the society waived the necessity of an Ethics Committee approval.

### Statistics

Data analysis was performed using the SPSS software package (SPSS 15.0, SPSS Inc., Chicago, IL, USA). Data are given as percentage of the total number of included questionnaires if not otherwise stated. For questions with multiple possible answers, each answer was analysed separately in regard of the percentage of positive and negative responses. Due to the descriptive character of the study, no p-values were computed.

## Results

**Figure 1:**
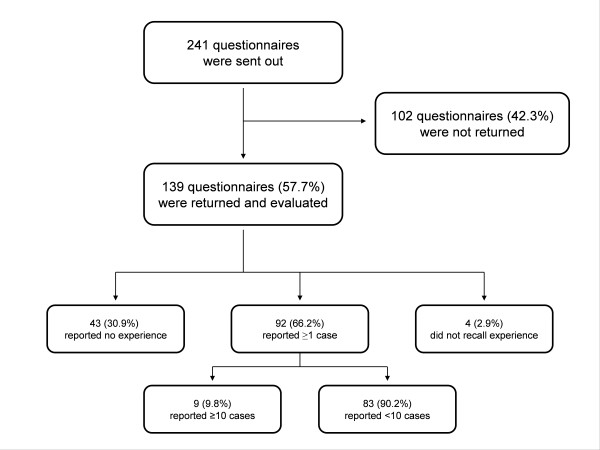
**Flow Chart**. 58% of the medical directors of all intensive care units in Austria completed the survey.

There were 241 questionnaires sent and 139 were turned, which was a response rate of 58%. Of all the returned questionnaires, 62% were answered by specialists for anaesthesiology and intensive care medicine and 30% by specialists for internal medicine. The median number of ICU admissions per year was 400 and the median number of ICU mortality was at 10%. About one third of the responders (31%) reported having had no experience with advance directives and only 9 directors of intensive care units (10%) had dealt with more than 10 advance directives in the previous two years (Figure 1).

The existence of a directive was either communicated by the patients themselves (28%) and/or by their relatives (77%) and/or the doctors actively searched for it (23%).

**Figure 2:**
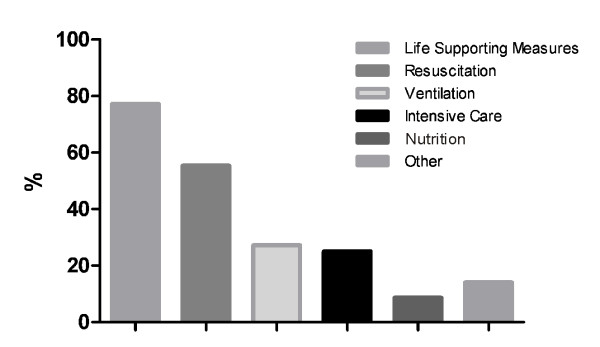
**Refused therapies**. Percentage of different therapies refused in advance directives.

**Figure 3:**
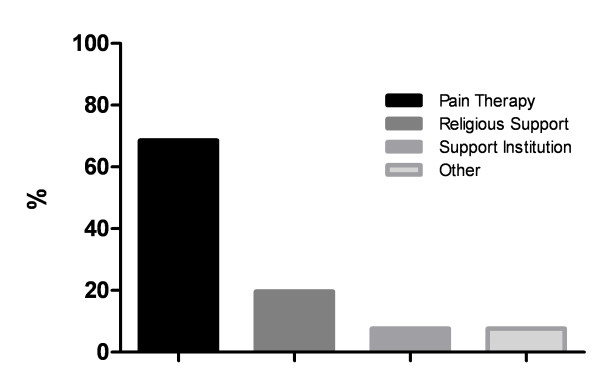
**Desired therapies**. Percentage of different therapies asked for in advance directives.

The percentage of refused and desired therapies is shown in Figure 2 and 3.

**Figure 4:**
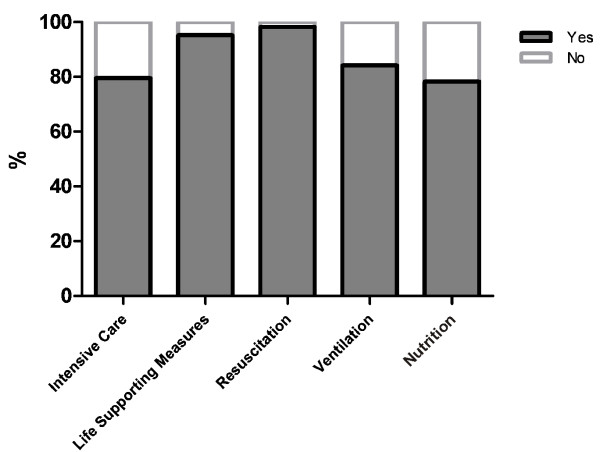
**Adherence to advance directives**. Percentage of positive ("Yes") and negative ("No") adherence to advance directives by physicians regarding intensive care, life-supporting measures, resuscitation, ventilation and nutrition.

Regarding resuscitation and life supporting therapy, intensive care physicians adhered to the directives in almost all cases (99% and 95% respectively). If patients refused mechanical ventilation or nutrition, the percentage of compliance was 80% and 78% respectively (Figure 4).

Almost the half of the doctors (48%) reported that conflicts had arisen due to the existence of an advance directive: conflicts either owing to their own ethical values and/or within the treatment team and/or sometimes also with the patients' relatives.

In total, 61% of the intensive care physicians considered themselves to be sufficiently informed; about half of them (55%) felt this was due to their own initiative.

The different impacts of binding and non-binding advance directives seemed to be quite unclear, 22% could not even remember which kind of directive they had dealt with.

Only 1% of the responders believed that an advance directive cannot be revoked, all other responders were either informed about the patients' right to do so, or were not sure about the legal situation.

In total, 73% considered advance directives to be helpful; however, less than the half (47%) of the physicians actively recommended the creation of a directive in general. Only 9% stated to have a directive themselves.

## Discussion

The main finding of this survey is that 2 years after enactment of the respective legislation, only about two-thirds of the directors of intensive care units in Austria have already dealt with advance directives. Our results are just in line with the findings of comparable studies done in the U.S. [8] and the Scandinavian region [9].

Nonetheless, a response rate of almost 60% proves the great interest of intensive care professionals in making patient-oriented end-of-life decisions. However, as long as patients do not make use of their right of co-determination, the mere existence of a legal framework for the creation of advance directives solves only part of the problem.

The Austrian law of 2006 comprises detailed guidelines for the creation of an advance directive. In order to make it binding, patients have to seek medical and legal advice - a procedure that is not only time-consuming, but also costly (up to € 500,-). Especially a binding advance directive has to include a detailed explanation of each possible medical intervention with regard to a concrete situation. Hence, an exact determination covering all possible circumstances is often difficult to achieve. In any case, people who create an advance directive have to reflect on their own mortality and, often more distressful, the possibility of their once becoming care-dependent. Therefore, advance directives are mainly created by people who are deeply concerned about their sovereignty in case of care dependence, e.g. patients suffering from chronic illnesses. A current qualitative research shows that patients in Austria use the law on advance directives in different ways to deal with questions of discontinuation of treatment [10]. The patients take into account not only their experiences with the medical system, but also their social situation and often try to use advance directives as a means of continuing communication in a situation when they cannot express themselves anymore. Three possible ways of interpreting advance directives have been identified: either as a safeguard or as a defence instrument or as a means of making the experience of dying somewhat less dreadful [10].

Particularly young and healthy people in Western European societies often see no reason to think or care about dying [11]. Only individual cases like Terry Schiavo in the U.S. or, recently, the 19-year-old Eluana Englaro in Italy make them aware of their own limited lifespan, at least for some time. What can be generally observed, is a problematic contradiction between the public "orchestration" of dying, as it occurred in the cases of Schiavo and Englaro, and the predominate model in modern societies of suffering and death as taboo.

As opposed to the recent, very emotional debate in Germany (finally the German Federal Parliament passed the according law in June 2009), the enactment of the advance directive legislation in Austria was not subject to public discussion. Hence, a misleading mix up with euthanasia, comparable to the recent dispute in Italy about the death of Eluana Englaro [12], could be avoided. On the other hand the low degree of interest and awareness may have contributed to the hitherto insufficient application of the law. Our study did not reveal any new points of criticism apart from the already mentioned difficulties in having to describe a wide variety of concrete end-of-life situations and the high costs arising from the creation of an advance directive.

Interestingly, less than 50% of the intensive care physicians (who are confronted with the narrow margin between life and death every day) recommend creating an advance directive. Results of a German study show that this attitude largely mismatches the expectations of the patients [13], of whom 80% expected their attending physician to address the issue of advance directives prior to an operation. Consequently, physicians should reflect more on the question as to when advance directives ought to be discussed with their patients. In a study done by Nicolasora and colleagues it was demonstrated that patients, who are well informed will very well determine their therapies [14]. In order to minimize misinformation the consultation should be done by intensive care physicians as the specialists in the field. As proposed in a French study, also general practitioners, who usually are closer connected to the patients and their families could participate in such meetings [15].

According to the results of our study, relatives are integrated into the decision-making process, but from a legal point-of-view relatives in Austria are denied the right to decide for the patient, unless clearly stated in an advance directive. Furthermore, as shown by the examination of Li et al, the appropriateness of surrogate decision has to be questioned [16]. The involvement in decision-making poses a major challenge to relatives [17,18]. Early determination of views and wishes regarding end-of-life care would most probably improve the communication and understanding between patients and their relatives regarding this often controversial topic. Conflicts not only between patient and relatives, but also between the medical team and relatives can thus be dispelled at an early stage by assuring a patient-oriented treatment, providing for the best possible understanding and acceptance of the relatives in an emotionally extreme situation.

Only 73% of the intensive care physicians were found to consider advance directives as helpful in their decision-making process. Our study does not show their motives in detail, so it remains unclear, whether e.g. some colleagues still prefer to decide according to their own ethical concepts instead of honouring their patients' wishes. Furthermore, another crucial aspect should be considered: If "Allow Natural Death" decisions become routine, mortality rates will maybe increase. However, as a consequence of therapy limitation, palliatively treated patients will not have the highest illness severity or therapeutic intervention severity scores. In this context, the evaluation of an intensive care unit's performance by assessing and comparing mortality rates should eventually be reconsidered, as otherwise some intensive care physicians might be misled to sustain life in order to decrease the mortality rate of their unit.

This survey has some limitations.

Results are only valid for Austria and cannot be transferred to other countries.

The physicians had to recall their experience with advance directives during the previous 2 years. Hence, their answers might be biased by basing on their memory and not their recent experience. A mix up of different patients, especially in correlation with the questions about refused and desired therapies, cannot be ruled out. Although the response rate was above average [8,15], still about one third of the ICU directors did not participate. Therefore, the survey might not reflect the real position of the whole community of physicians regarding advance directives. Although performing structured interviews might be a more efficient method to obtain clear and detailed answers on such a delicate issue, we decided to create a questionnaire in order to reach intensivists from all over the country.

## Conclusion

Confronted with end-of-life decisions Austrian ICU physicians are very much concerned about integrating their patients' preferences into their decision making process. However, as long as patients do not make use of their right of co-determination, the enactment of the new law can be considered only a first important step forward.

## Key Messages

✓ 2 years after enactment of the respective legislation, only about two-thirds of the directors of intensive care units in Austria have already dealt with advance directives.

✓ Adherence to an advance directive may cause conflicts; either owing to the physicians' ethical values or within the treatment team or with the patients' relatives.

✓ As long as patients do not make use of their right of co-determination, the mere existence of a legal framework for the creation of advance directives solves only part of the problem.

## Competing interests

The authors declare that they have no competing interests.

## Authors' contributions

ES created the questionnaire, organised the survey, collected the data and drafted the first version of the manuscript. PH contributed considerably to the Discussion section bringing in her expertise as a publicist. AS critically revised the manuscript with particular attention to the legal background. SH participated in the study design and performed the statistical analysis. CK participated in the study design and coordination and helped to draft the manuscript. All authors read and approved the final version of the manuscript.

## Pre-publication history

The pre-publication history for this paper can be accessed here:

http://www.biomedcentral.com/1472-6939/11/19/prepub

## Supplementary Material

Additional file 1 **Questionnaire**. Translated questionnaire consisting of 21 questions. Click here for file
