# Supplementary material for: The role of advance directives in end-of-life decisions in Austria: survey of intensive care physicians
Source: BMC Med Ethics. 2010 Oct 21;11:19. doi: 10.1186/1472-6939-11-19 (PMC2976730; doi:10.1186/1472-6939-11-19)
Supplement: Additional file 1 — Questionnaire. Translated questionnaire consisting of 21 questions. [file 1472-6939-11-19-S1.DOC]

**Questionnaire**

1 Have you treated patients with an advance directive?

Yes

No

Not memorable

2 If yes, how often?

More than 10 times

Less than 10 times

3 How did you come to know about the advance directive?

I asked/searched for it

The patient gave it to me

The relatives gave it to me

Different (space for comment)

4 Which form of advance directive was predominant?

Binding

Non-binding

Not memorable

5 Which therapies were refused?

Intensive care

Life-supporting measures

Resuscitation

Ventilation

Alimentation

Different (space for comment)

6 Which special treatments were requested?

Pain Therapy

Residential home

Religious support

Different (space for comment)

7 Did you adhere to the advance directive?

Yes/No for every single therapy listed above

Space for comment

8 Did you check the date of the advance directive?

Yes

No

Not memorable

9 Did you encounter any conflicts related to the advance directive?

With own values

Within the team

With the relatives

Different (space for comment)

10 Are you sufficiently informed about the current law?

Yes

No

Don´t know

11 What are your sources of information regarding advance directives?

Employer

Medical societies

Own initiative

Different (space for comment)

12 Can an advance directive be revoked?

Yes

No

Don´t know

13 If yes, who can revoke it?

Patient himself

Relatives

Attending physician

14 Is an advance directive supporting your treatment?

Yes

No

Don´t know

15 In general, do you recommend the creation of an advance directive?

Yes

No

Don’t know

16 Do you have an advance directive?

Yes

No

17 Did the new law change your treatment behavior?

Yes

No

Don´t know

18 Which specialty does your ICU belong to?

Anesthesia

Internal Medicine

Surgery

Different (space for comment)

19 How many patients do you treat per year?

20 What is the mortality rate at your ICU?

21 Who is involved in end-of-life decisions at your ICU?

Patient himself

Medical director of the ICU

Physicians

Nurses

Relatives

Ethics committee

Different (space for comment)
